# Plasmacytoid urothelial carcinoma of the bladder: a case report

**DOI:** 10.1186/1757-1626-2-6647

**Published:** 2009-04-28

**Authors:** Saad Aldousari, Kanishka Sircar, Wassim Kassouf

**Affiliations:** 1 Division of Urology, McGill University HealthCenter, 1650 Cedar Avenue, Rm L8-315, Montreal, Quebec, Canada; 2 Division of Pathology, McGill University HealthCenter, 1650 Cedar Avenue, Rm L8-315, Montreal, Quebec, Canada

## Abstract

Plasmacytoid bladder cancer is a rare variant of transitional cell carcinoma. A 57-year-old man was referred to our institution for management of invasive transitional cell carcinoma diagnosed at a peripheral hospital. His complaints were of vague lower abdominal pain with associated urgency and frequency requiring oxybutynin. Metastatic workup was negative and was subsequently scheduled for a radical cystectomy. Routine colonoscopy 3 weeks prior to surgery was negative. Intraoperatively, he was found to have metastatic urothelial cancer involving the cecum and multiple metastatic deposits within the mesentery of the small intestines. He underwent a palliative cystectomy with ileal conduit formation. Final pathology revealed metastatic plasmacytoid variant of urothelial cancer. Histology and immunohistochemistry were compatible with plasmacytoid variant of urothelial cancer. Here we present our case of this rare variant of urothelial cancer with a review of its characteristics.

## Introduction

Bladder cancer is the 4^th^ most common malignancy in males. It most commonly exists as epithelial tumor where 90% of cases are transitional cell carcinoma with a papillary appearance. In recent decades multiple variants of this epithelial tumor have been described, the significance of which lies in the impact it has on prognosis and approach to management. We describe plasmacytoid variant of transitional cell carcinoma in a 57-year-old man who was referred to our institution for further management. Less than 30 cases have been described in the literature, and almost all were invasive and metastatic.

## Case Presentation

**Figure 1:**
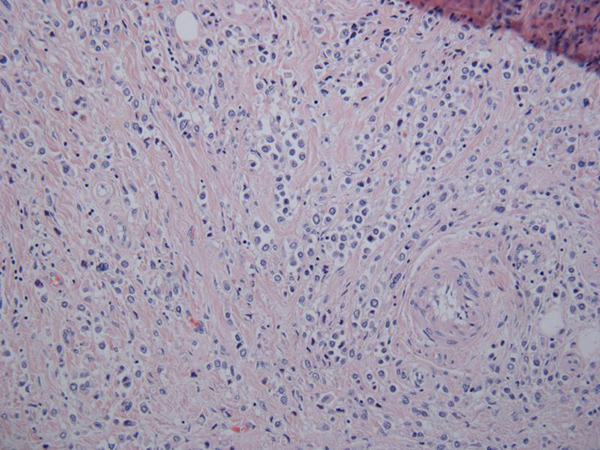
**Urothelial carcinoma with plasmacytoid features on low power**.

**Figure 2:**
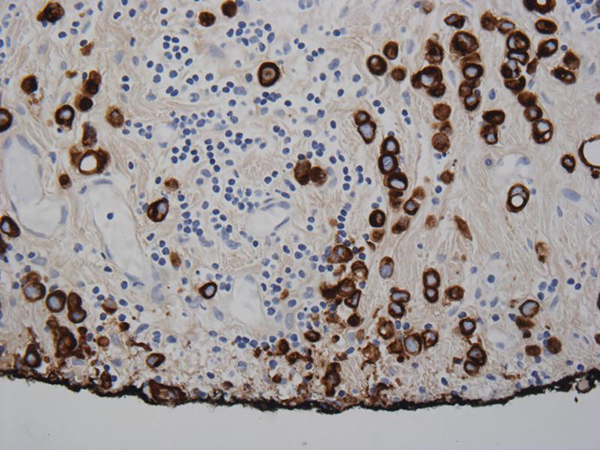
**Keratin positive margin of urothelial mucosa with transitional cell carcinoma**.

**Figure 3:**
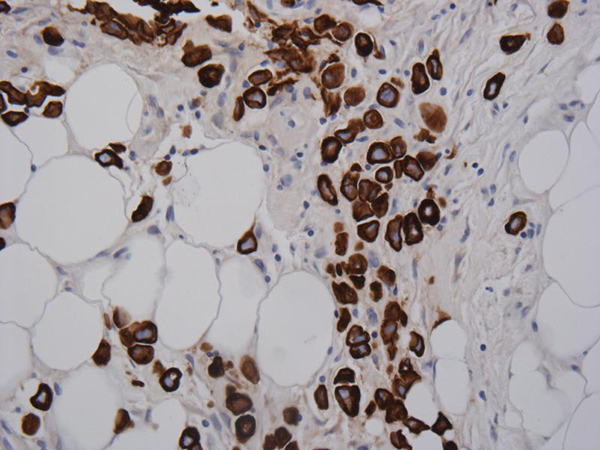
**Mesenteric nodule with keratin positive immunostaining of metastatic urothelial transitional cell carcinoma**.

A 57-year-old French Canadian male presented with gross hematuria and worsening lower urinary tract symptoms. He was known for a history of recurrent superficial TCC refractory to two induction courses of BCG. He had a 35-pack year history of smoking and no family history of genitourinary malignancy. Cystoscopy revealed abnormally 'edematous appearing' mucosa in the posterior wall of the bladder. Urine sent for cytology was negative. Metastatic workup including CT chest/abdomen/pelvis and colonoscopy were negative and was subsequently scheduled for a radical cystectomy. Exploratory laparotomy revealed multiple metastatic lesions involving the GI tract and mesentery. Frozen sections were sent which were consistent with metastatic poorly differentiated TCC. Since patient was significantly symptomatic, he underwent a palliative cystectomy with ileal conduit diversion. Final pathology revealed muscle-invasive high-grade urothelial carcinoma of the bladder with plasmacytoid features penetrating through the entire bladder wall and into the serosa (Figure 1). Immunostaining was positive for cytokeratin, confirming plasmacytoid urothelial carcinoma (Figure 2). The metastatic lesions were also positive for plasmacytoid features (Figure 3). The pathological stage of urothelial cancer was pT3a, N0, M1. The patient had an uneventful postoperative recovery. He was discharged 10 days post operatively. He received postoperative systemic chemotherapy (gemcitabine/cisplatin); however, he passed away within 6 months due to rapid progression of disease.

## Discussion

Plasmacytoid urothelial carcinoma is an extremely rare bladder cancer variant. Sahin et al described the first case in 1991 [1]. Since then there have been 24 cases described in the literature in the form of case reports or case series [2]. This tumor variant arises from the bladder urothelium as opposed to metastatic or inflammatory infiltration with plasma-like cells. Histologically, these cells appear in sheets or single cells infiltrating the lamina propria and muscularis mucosa with a high potential for invasion and metastasis. Most cases described were high grade and poorly differentiated with metastasis at presentation. Cancer cells display an eccentric nuclei and poorly identified nucleoli. They have an eosinophilic cytoplasm and a stroma with a predominantly myxoid quality. Two reports in the literature described the presence of cells resembling signet ring cell carcinoma confirmed by mucin positivity [3,4]. The plasmacytoid urothelial carcinoma can coexist with non-invasive papillary urothelial cancer [5], carcinoma in situ [6,7], or invasive high grade urothelial carcinoma. One recent report describes a case of infiltrative plasmacytoid carcinoma of the bladder in a TURBT specimen which after cystoprostatectomy demonstrated pure plasmacytoid cells with no urothelial cancer present [8].

When the histological appearance is highly suspicious for this variant of urothelial carcinoma, immunohistochemistry is a key for making the diagnosis. Looking like plasma cells, these cells also stain positively for CD-138, a plasma cell marker. But they also stain positively for epithelial markers like cytokeratin and epithelial membrane antigen but not for hemopoietic markers like CD-79a, kappa and lambda light chain. A recent case report by Shimada et al was the first to show urothelial carcinoma with a plasmacytoid variant producing both CA19-9 and βHCG in the absence of trophoblastic tissue [9].

Treatment of this variant is very difficult owing to the late presentation with metastases and lack of data on its response to systemic chemotherapy. Interestingly, Khono and associates recently reported the first pathological complete response found at radical cystectomy for cT4N0M0 plasmacytoid urothelial carcinoma treated with neoadjuvant chemotherapy (methotrexate, vinblastine, etoposide, and cisplatin) [7].

## Conclusion

Plasmacytoid urothelial carcinoma is an extremely rare variant of bladder cancer and is associated with dismal prognosis. Due to its aggressive nature, multimodal therapy should be considered; however, the response to neoadjuvant chemotherapy needs to be further evaluated.

## Abbreviations

BPH: Benign Prostatic Hyperplasia; CT: Computerized Tomography; TCC: Transitional cell carcinoma; TURBT: Transurethral Resection of Bladder Tumor; BCG: Bacillus Calmette-guerin; GI: Gastro Intestinal; βHCG: Beta Human Chorionic Gonadotrophin.

## Consent

As previously communicated, we were not able to obtain consent since the patient is deceased and has no relatives alive at the present time.

## Competing interests

The authors declare that they have no competing interests.

## Authors' Contributions

SA did the literature search on plasmacytoid urothelial carcinoma and analyzed and interpreted the patients' data and chart. SA also was a major contributor in writing the manuscript. WK is the principal surgeon of the patient and was a major contributor in writing the manuscript.
